# 16-O-methylcafestol is present in ground roast Arabica coffees: Implications for authenticity testing

**DOI:** 10.1016/j.foodchem.2017.12.034

**Published:** 2018-05-15

**Authors:** Yvonne Gunning, Marianne Defernez, Andrew D. Watson, Niles Beadman, Ian J. Colquhoun, Gwénaëlle Le Gall, Mark Philo, Hollie Garwood, David Williamson, Aaron P. Davis, E. Kate Kemsley

**Affiliations:** a Analytical Sciences Unit, Institute of Food Research, Norwich Research Park, Norwich NR4 7UA, UK; b Oxford Instruments, Tubney Woods, Abingdon, Oxford OX13 5QX, UK; c Royal Botanic Gardens (RBG), Kew, Richmond, Surrey TW9 3AE, UK

**Keywords:** NMR, Spectroscopy, Low-field, Coffee, Adulteration, Species, Arabica, Robusta, Authentication

## Abstract

High-field and low-field proton NMR spectroscopy were used to analyse lipophilic extracts from ground roast coffees. Using a sample preparation method that produced concentrated extracts, a small marker peak at 3.16 ppm was observed in 30 Arabica coffees of assured origin. This signal has previously been believed absent from Arabicas, and has been used as a marker for detecting adulteration with robusta. Via 2D 600 MHz NMR and LC-MS, 16-O-methylcafestol and 16-O-methylkahweol were detected for the first time in Arabica roast coffee and shown to be responsible for the marker peak. Using low-field NMR, robusta in Arabica could be detected at levels of the order of 1–2% w/w. A surveillance study of retail purchased “100% Arabica” coffees found that 6 out of 60 samples displayed the 3.16 ppm marker signal to a degree commensurate with adulteration at levels of 3–30% w/w.

## Highlights

• Lipophilic extracts of ground roast Arabica coffees were authenticated by benchtop NMR.
• Small amounts of esterified 16-O-methylcafestol were found in Arabica coffees.
• The compound identity was confirmed by NMR and MS experiments.
• 16-OMC remains a useful marker for non-Arabicas as these contain much higher amounts.
• 6 out of 60 retail Arabicas contained significant amounts of non-Arabica species.

## 1 Introduction

Coffee is a major tropical agricultural crop, and one of the most widely traded global commodities (International Coffee Organization., 2017b). Of the 124 species known to science (Davis et al., 2006, Davis et al., 2011), only two are commercially important: *Coffea arabica* L. (Arabica coffee) and *C. canephora* Pierre ex A. Froehner (robusta, or conilon) (Belitz, Grosch, & Schieberle, 2009). Although generally more difficult to grow than robusta, Arabica represents around 60% of global production.

Due to the superior organoleptic properties of the roasted beans, Arabica coffees command a higher price than robusta. The opportunity for fraudulent economic gain by substituting Arabica with robusta beans is therefore obvious (Toci, Farah, Pezza, & Pezza, 2016). The identity of intact beans can be verified by inspection (International Coffee Organization, 2017b, Mendonca et al., 2009), but for ground roast products some form of chemical assay becomes necessary.

In a recent paper (Defernez et al., 2017), we reported the use of low-field (60 MHz) ^1^H NMR spectroscopy to screen lipophilic extracts from ground roast coffees for the undeclared presence of robusta in products labelled “100% Arabica”. Our work exploited a marker compound, the diterpene 16-O-methylcafestol (16-OMC). When present in coffee, 16-OMC is mostly present in esterified form and exhibits a resonance in the ^1^H NMR spectrum at 3.16 ppm that is well-resolved and isolated from other signals even at 60 MHz. Esterified 16-OMC is a well-documented minor compound of robusta beans, and as long ago as 1989 was reported as absent from Arabica coffee (Speer & Mischnick, 1989). Since then the prevailing literature consensus has reiterated its absence from Arabica coffees (Bonnlander et al., 2007, de Roos et al., 1997, Kamm et al., 2002, Kurzrock and Speer, 2001, Pacetti et al., 2012, Pettitt, 1987, Speer and Kölling-Speer, 2006). It has therefore been used as a marker for the presence of robusta in coffee products, including in a recognized method for authenticity testing (DIN 10779., 2011).

In our previous work, we described using the 3.16 ppm peak area as a proxy for the amount of robusta coffee present in a sample. Our estimated detection limit was around 10% w/w robusta in Arabica. In our new research, presented here, chloroform is again used to extract the lipophilic phase from a sample of ground roast coffee, but in contrast to our previous approach, this is followed by a concentration step: the chloroform is evaporated using a vortex evaporator and the residue dissolved in a much smaller amount of relatively higher-cost deuterated chloroform. This allows the use of much larger amounts of coffee and solvent in the extraction step, without the method becoming prohibitively expensive – an important consideration for a putative screening technique. The concentrated extract produces an NMR spectrum with larger peaks and greater signal-to-noise from a given set of acquisition conditions. This is especially helpful with regards to detection of minor compounds such as esterified 16-OMC. As a result of this improvement, we are now attaining a detection limit of the order of 1% w/w robusta in Arabica. This is an important milestone. Although there is no universal statutory definition, food fraud is generally accepted to mean intentional substitution or adulteration of food products for economic gain, rather than simply adventitious contamination during normal processing. It has been proposed that for many products, 1% w/w of adulterant is a reasonable cut-off for making this distinction (Food Standards Agency., 2013), although this will depend on the commodity and normal production practices in the sector. With regards to the undeclared presence of robusta in Arabica, even substitution at a rate of a few percent could yield substantial economic advantage, considering the price differential between the two species and the amount of coffee traded.

An unexpected result of the improvement in limit of detection was the surprising discovery of low levels of 16-OMC in Arabica coffees, in contrast to previous studies. Confirmation of this finding was sought using a range of Arabicas of assured provenance. Throughout, the 60 MHz results were cross-validated by comparison with 600 MHz ^1^H NMR. LC-MS and 2D NMR were also used to confirm the annotation of the 3.16 ppm resonance.

Finally, the new preparation procedure was used to conduct a surveillance exercise of declared “100% Arabica” ground roast coffee samples sourced from a range of retailers around the world. The aim was to look for evidence of samples adulterated with robusta or other coffee species, and estimate the prevalence of this kind of fraud in the sector.

## 2 Materials and methods

### 2.1 Samples

#### 2.1.1 Samples used in method improvement and development work

Three samples of roast coffee beans (2 Arabica, 1 robusta) were obtained locally (trusted UK commercial suppliers, British Coffee Association). These samples were used in the development of the sample preparation procedure.

Two of these (one Arabica, one robusta) were used to prepare a series of 18 mixtures, with the proportion of robusta covering the range 0–16% w/w, to facilitate direct comparison with our previously reported calibration results. The other Arabica was paired with the robusta and used to prepare a further 9 samples, at robusta contents of between 1 and 24% w/w. Both series were used in a simulated authenticity test based on the samples of assured origin described below (Full details of the mixture samples are included in Supplementary Fig. 5).

#### 2.1.2 Arabica and “non-Arabicas” of assured origin

Forty samples of green coffee beans were obtained by in situ collection from coffee farms/farming districts, or from samples of known provenance (Royal Botanic Gardens (RBG), Kew). Thirty of these were Arabica coffees, of which 18 were wild types (17 Ethiopian, 1 Colombian). The Ethiopian wild types were collected directly from coffee farms (by either A.P. Davis, or other staff at the RBG). Numerous genetic analyses (Tadele et al., 2014, Tesfaye et al., 2007, Tesfaye et al., 2014) show that Ethiopian cultivated stock is derived directly from the wild *C. arabica* genepool, with no indications of hybridization with robusta (*C. canephora*), or Arabica cultivars backcrossed with robusta (e.g. Coffee cv. ‘Catimoor’). Moreover, robusta coffee is essentially absent from Ethiopia; it occurs in neighbouring countries (southern South Sudan, and Kenya), but thousands of kilometers from wild or farmed Arabica in Ethiopia. There is a very small amount of robusta grown in the far south west in Bebeka (Bench Maji), but this is a considerable distance from the origin of any of the samples examined here. The remaining 12 Arabica samples were cultivars from a range of commercially important coffee producing countries. The final 10 assured origin samples were “non-Arabica” species (6 *C. canephora*, 2 interspecies hybrids, 2 Liberica).

The Arabica samples were from the harvest years 2014–2016; the non-Arabica samples from 2013. All samples were roasted to a medium roast profile (IKAWA Pro Sample Roaster, Ikawa Ltd., London) before sample preparation. Complete details of these samples are given in Supplementary Table 1.

#### 2.1.3 Samples for quantitative work

One of the authentic Arabica samples (sample 12) was used to determine limit of detection (LoD) and limit of quantitation (LoQ); for this, a series of 8 extracts spiked with different amounts (0, 0.025, 0.05, 0.1, 0.2, 0.3, 0.4, 0.5 mg/ml in chloroform-d) of 16-OMC (Sigma-Aldrich, formula depicted in Fig. 3) was prepared. The LoD and LoQ were also determined using a modification of a previously described method (Schievano, Finotello, De Angelis, Mammi, & Navarini, 2014) employing a series of mixtures in which the same Arabica sample was spiked at different levels (25, 50, 80, 90%) with a commercial robusta sample. The chloroform-d used to dissolve the dried extracts (Section 2.2) contained 2.5 mM DMF as an internal concentration standard.

#### 2.1.4 Survey of retail coffees sourced worldwide

60 samples of ground roast coffees were purchased by IFR staff, students and collaborators from a range of outlets in 11 different countries. All displayed the labelling claim “100% Arabica” or equivalent, in the relevant local language. The geographic origins of the coffees as stated on the labels covered 11 different coffee-growing countries and represented all producing continents. Details of each sample are given in Supplementary Table 2. All samples were supplied to IFR's Analytical Sciences Unit in original unopened packaging.

### 2.2 Sample preparation

All whole bean coffee samples were ground using a Braun coffee grinder which was thoroughly cleaned between the grinding of each sample. Retail-purchased ground roast coffees were prepared directly from the pack, with no further grinding step. Gradation tests (0.1, 0.3, 0.5 and 1 mm sieves) determined that ground sample particle sizes were typically distributed across the range 0.3–1 mm for both purchased ground roast and in-house ground coffees.

The lipophilic fraction was extracted by taking 10 g of ground sample and stirring (600 rpm) with 30 ml of chloroform for 5 min. The extract was filtered through filter paper (Whatman No. 1). It was then put through an empty SPE cartridge (Bond Elut) configured for simple filtration (20 μl polyethylene frits) into sovirel tubes. The extract was dried using a vortex evaporator with heating at 30 °C and a pressure of 30 in Hg for 30 min. The dried extract was redissolved in 800 µl of chloroform-d and filtered through cotton wool directly into 5 mm NMR tubes. For the quantitative work, recovery of the lipophilic phase in chloroform was recorded, amounting to 8 ± 0.5 ml.

A duplicate extraction procedure was carried out for a number of samples as indicated in the discussion.

### 2.3 Spectral acquisition

#### 2.3.1 Low-field ^1^H NMR spectroscopy

60 MHz ^1^H NMR spectra were acquired on a Pulsar low-field spectrometer (Oxford Instruments, Tubney Woods, Abingdon, Oxford, UK) running SpinFlow software (v1, Oxford Instruments). The sample temperature was 37 °C, and the 90° pulse length was 13.28 μs as determined by the machine’s internal calibration cycle. For each sample, 256 free induction decays (FIDs) were collected using a filter width of 5000 Hz, acquisition time of 6.55 s and recycle delay of 2 s, resulting in a total acquisition time of approximately 40 min per extract. These parameters represent an acceptable compromise between speed and spectral quality. FIDs were zero-filled to give spectra of 65,536 points. The linewidth was maintained between 0.5 and 0.9 Hz by daily checking of the chloroform FWHM and shimming as and when necessary.

In all cases, the FIDs were Fourier-transformed, co-added and phase-corrected using SpinFlow and MNova (Mestrelab Research, Santiago de Compostela, Spain) software packages to present a single frequency-domain spectrum from each extract. The chemical shift scale in all spectra was referenced to the residual chloroform peak at 7.26 ppm.

#### 2.3.2 High-field ^1^H NMR spectroscopy

600 MHz ^1^H NMR spectra were collected from selected extracts using a Bruker Avance III HD spectrometer running TopSpin 3.2 software and equipped with a 5 mm TCI cryoprobe. The probe temperature was regulated at 27 °C. For each spectrum 64 scans were collected using the *zg30* sequence with P1 = 1 µs (pulse length 0.33 µs equivalent to a pulse angle of 4°), a spectral width of 20.5 ppm, acquisition time of 2.67 s and relaxation delay of 3 s. Total acquisition time was 6 min. The receiver gain was adjusted automatically for each sample prior to acquisition to avoid receiver overload. FIDs were zero-filled and transformed using exponential line broadening (0.3 Hz) to give spectra of 65,536 points. The spectra were referenced to the residual chloroform peak at 7.26 ppm. For the quantitative spiking experiments the receiver gain was fixed at 28.5 and the relaxation delay could be maintained at 3 s because of the extremely low pulse angle. It was found that the intensity ratio of the 3.16 ppm signal to the DMF methyl signal (2.95 ppm) was not changed by increasing the relaxation delay to 43 s, the value used previously (Schievano et al., 2014).

Heteronuclear Single Quantum Coherence (HSQC) and Heteronuclear Multiple Bond Coherence (HMBC) spectra were run using standard Bruker pulse sequences with 2048 (t_2_) × 256 (t_1_) data points, zero-filled to 2048 × 1024 points on Fourier transformation. The ^1^H × ^13^C spectral widths were 12 × 165 ppm (HSQC) and 12 × 250 ppm (HMBC) and the number of scans per t_1_ increment (NS) was for the Arabica sample NS = 1152 (HSQC), NS = 960 (HMBC) and for the robusta sample NS = 4 (HSQC), NS = 128 (HMBC).

### 2.4 NMR data analysis

All data visualization and processing of the frequency-domain spectra was carried out using Matlab (The Mathworks, Cambridge, UK) installed along with the 'Statistics and Machine Learning' and 'Signal Processing' toolboxes, making use of a range of inbuilt functions.

To visualize or quantify individual peaks or groups of peaks, the relevant region was locally baseline corrected using second order polynomial fitting.

Wherever direct comparisons were made between low-field and high-field data, spectra were first internally normalized through division by the integrated glyceride region (3.9–4.6 ppm) (Parker et al., 2014). This mitigates for variations in overall intensity; this can arise from slight changes in sample concentration due to evaporation between the time of acquisition of the low- and high-field spectra, and also from differences in the receiver gain setting which was optimized for each spectrum individually on the high-field instrument in order to avoid baseline artefacts.

Simple linear regression with an intercept term was used to model the sample robusta concentration as a function of integrated area of the 3.16 ppm peak, in both raw and normalized spectra as stated.

### 2.5 Mass spectrometry analysis

Ultra high performance liquid chromatography – time of flight mass spectrometry was performed with a Waters Acquity UPLC interfaced to a Waters Synapt G2-Si QTOF mass spectrometer (Herts, UK). The chromatography column was a Waters HSS T3 C18 capillary column (100 × 2.1 mm, particle size 1.7 µ) maintained at 40 °C with a flow rate of 400 µl/min. The reversed phase gradient profile consisted of a binary gradient initially of 95% A (water + 0.1% formic acid (Greyhound Chemicals, Merseyside, UK)) and 5% B (acetonitrile (Chromasolv LC-MS grade, Fluka, Poole, UK) + 0.1% formic acid) increasing to 95% B at 41 min (see Supplementary Fig. 3 for details). Water was in-house Millipore ultra-distilled. The injection volume was 5 µl.

The mass spectrometer was run in positive ion electrospray mode with a ‘single stage’ (zero collision energy) *m*/*z* selection. The scan time was 0.3 scan/s and the mass range 50–1200 Da. The mass accuracy was 20,000 FWHM (±0.005 Da).

Ground powders (0.2 g) were saponified with 2 ml of 2.5 M KOH in EtOH (Fluka) at 80 °C for 1 h as set out in de Souza & Benassi, (2012). Work-up was as described except that diethyl ether (Sigma, Poole, UK) rather than *t*-butyl methyl ether was used to extract the organic phase. The dried organic phase was resuspended in 1.5 ml 20% MeCN.

## 3 Results and discussion

### 3.1 Development work: The effect of the revised sample preparation method

**Fig. 1:**
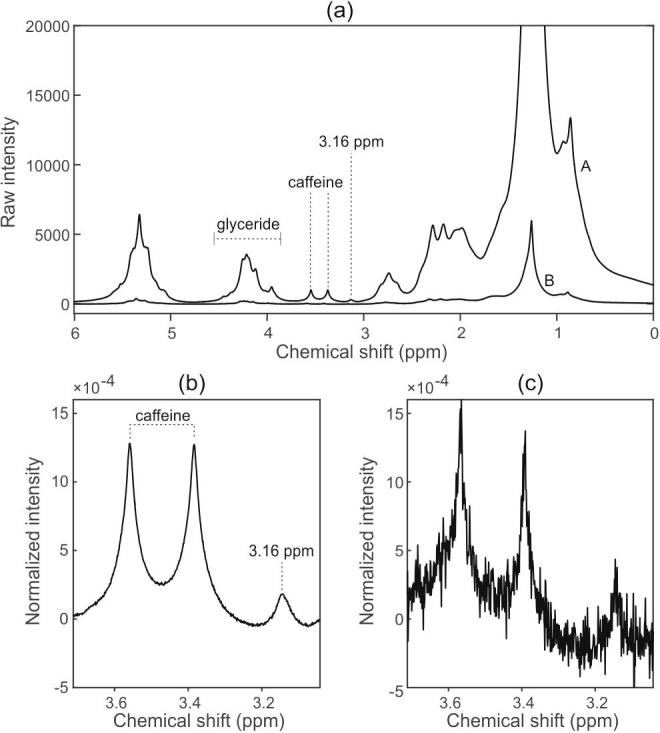
(a) 60 MHz ^1^H NMR spectra obtained from two lipophilic extracts prepared from a sample of robusta coffee beans. Spectrum A is of an extract prepared using the method involving a concentration step; spectrum B is of an extract prepared using the previously reported direct extraction method. Panels (b) and (c) show expansions around the 3.16 ppm and caffeine peaks (∼3.38, ∼3.58 ppm), for the concentrated and direct methods respectively.

Fig. 1(a) shows the 60 MHz spectra obtained from two lipophilic extracts prepared from a sample of robusta coffee beans. The data are presented as raw intensities, after co-addition and zero-filling but with no further processing such as apodization. Spectrum A is of an extract prepared using the method described herein, which involves a concentration step; spectrum B is of an extract prepared using the direct extraction method we used in our previous work (Defernez et al., 2017). The difference in overall spectral intensity reflects the concentration difference resulting from the two preparation methods.

Fig. 1(b) and (c) show the region of interest around the 3.16 ppm peak with and without the concentration step, respectively. This peak is conventionally attributed to the H21 methyl protons in esterified 16-OMC (Scharnhop and Winterhalter, 2009, Schievano et al., 2014). The plot also shows neighbouring resonances from caffeine, included to give a sense of scale. Here, the spectra have each been internally normalized to the area of the glyceride peaks at 3.9–4.6 ppm, to allow plotting on the same scale and direct side-by-side comparison. Fig. 1(b) and (c) show that the concentration step gives an improved-signal-to noise. We can conclude that this improvement is wholly due to the new sample preparation method, specifically the concentration step, since the spectra were collected using the same spectrometer and acquisition conditions, and the extracts were prepared from the same original coffee sample.

**Fig. 2:**
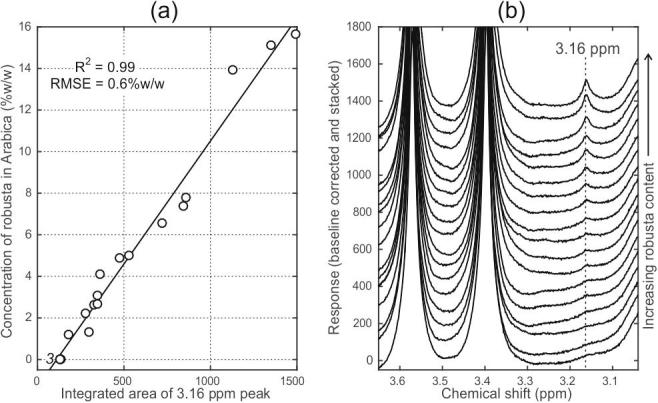
(a) Robusta content of a mixture series versus the integrated area of the 3.16 ppm peak in 60 MHz spectra; simple linear regression line is indicated. (b) The 3.16 ppm region from the mixture series spectra shown as a stacked plot for clarity. The three bottom traces are from repeat extractions of 100% Arabica beans.

This revised procedure also leads to a substantial improvement in the ability to calibrate for the robusta content in a series of robusta/Arabica mixtures. The linear relationship between the robusta content and the integrated area of the 3.16 ppm peak recorded at 60 MHz is demonstrated in Fig. 2(a) for the series of 18 mixture samples. Simple linear regression yields an R^2^ value of 0.99, and a root-mean-square error (RMSE) in the prediction of compositional values of 0.6% w/w. This compares highly favourably with analogous experiments we reported previously, which were carried out on mixture series prepared without a concentration step, for which the RMSE values were 7% w/w (Defernez et al., 2017).

Fig. 2(b) shows the 3.16 ppm region from the spectra of the entire series of mixtures, again with the neighbouring caffeine peaks included, and using a stacked plot for clarity. An unexpected finding is that the spectra from the 100% Arabica samples (bottom three traces) contain a tiny but consistent feature at 3.16 ppm. 600 MHz NMR spectra were acquired from the same series of extracts (Supplementary Fig. 1) which confirmed these findings.

### 3.2 Further investigation of the 3.16 ppm peak in Arabica samples

From the perspective of a ^1^H NMR-based authenticity test, the identity of the compound responsible for the 3.16 ppm peak is not as important as the fact that the peak exists at all, since it challenges the use of a simple ‘peak/no peak’ test for adulteration (Defernez et al., 2017). Nevertheless, because the peak is unforeseen and because other testing methodologies, for example HPLC or mass spectrometry, are independent of NMR peaks, it is appropriate to investigate what compounds could be responsible for this signal.

Detecting a peak at 3.16 ppm in Arabica is unexpected. One potential cause could be cross-contamination (carryover) from robusta samples to Arabica, particularly via the coffee grinder. To test for carryover, a fresh Arabica sample was ground from beans using an entirely new grinder. A 3.16 ppm peak consistent with earlier results on that sample was detected (data not shown). A series of alternating Arabica-robusta measurements, each time using freshly ground beans drawn from the same two samples, showed 3.16 ppm peaks in the Arabicas with a variation consistent with replicates without the interleaved robusta samples.

**Fig. 3:**
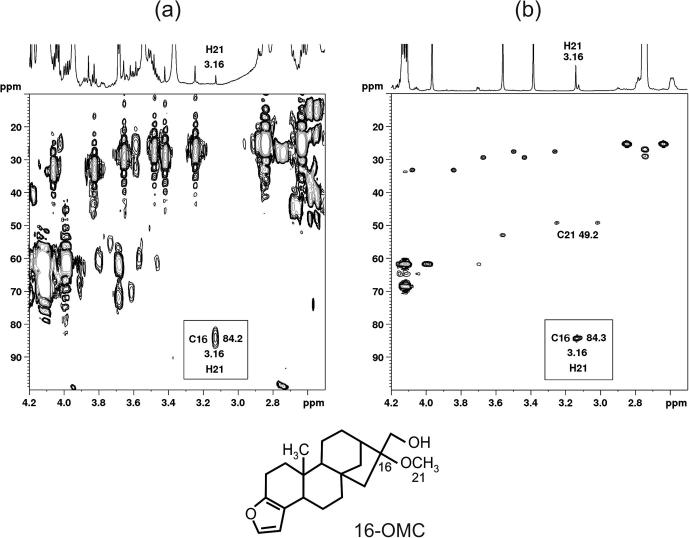
HMBC spectra of (a) an Arabica and (b) a robusta sample, together with the structure of 16-O-methylcafestol. The corresponding ^1^H spectra are shown above each plot with the 3.16 ppm peak indicated (H21 of esterified 16-OMC and 16-OMK). Note the difference in intensity: in (a) the peak immediately to the left of the 3.16 ppm signal, and slightly stronger, is the ^13^C satellite of one of the caffeine peaks; in (b) this satellite is not visible on the scale plotted. The cross peak highlighted at 3.16/84.2 ppm in both plots is the ^3^J_HC_ mediated correlation between H21/C16. No other ^1^H/^13^C correlations were detected on the 3.16 ppm trace. The direct ^1^J_CH_ correlation H21/C21 may also be seen (via the ^13^C satellites) in (b). The corresponding signals were too weak to be seen in (a) although the correlation was detected in the HSQC spectrum of Arabica.

To further examine the provenance of the residual peak at 3.16 ppm in Arabica coffees, a number of experiments were conducted using 2D high-field NMR. HSQC and HMBC experiments were carried out on a pure Arabica sample with a weak ^1^H signal at 3.16 ppm and, for confirmation, on a robusta sample in which the equivalent signal was much stronger. The HSQC experiment (data not shown) had a correlation peak in both samples at ^1^H/^13^C chemical shifts of 3.16/49.2 ppm. This is compatible with the assignments for H21/C21 signals in the spectrum of 16-OMC (Scharnhop and Winterhalter, 2009, Schievano et al., 2014) and of 16-OMK (Scharnhop & Winterhalter, 2009). In addition, in the HMBC experiment (Fig. 3), both samples displayed a signal at 3.16/84.2 ppm which is exactly as expected for the H21/C16 cross peak transmitted via the ^3^J_HC_ coupling in these two compounds (Scharnhop & Winterhalter, 2009). Furthermore no other ^13^C signals could be detected for the ^1^H trace passing through 3.16 ppm. This, together with the fact the chemical shifts do not match the other main diterpenes such as cafestol, kahweol or their dihydro versions, or other diterpenes that have also been found in some coffee species (four diterpenes without a H21-methyl group (de Roos et al., 1997)), make 16-OMC/OMK the most likely contributors to the 3.16 ppm marker peak. For robusta sample, inspection of the 3.16 ppm region in the 600 MHz ^1^H spectrum showed a very minor peak at 3.175 ppm in addition to the major peak at 3.164 ppm. These signals were assigned to the free and esterified forms of 16-OMC respectively (Schievano et al., 2014)). Additional minor signals at 3.147–3.152 ppm have been attributed to decomposition products of 16-OMC (Monakhova et al., 2015). These were not detectable in Arabica extracts where, of course, the main 3.164 ppm peak was much weaker than in robusta (see Fig. 3 and Supplementary Fig. 2). Identification of 1D-NMR signals which would distinguish between 16-OMC and 16-OMK is not possible in Arabica extracts; for instance, H1 and C1, which would differ most in terms of chemical shift, are hidden by other signals. It is also not easily feasible by 2D because there are much larger amounts of (esterified) cafestol and kahweol present, whose strong signals hinder the detection of signals from 16-OMC and 16-OMK.

For these reasons, a concise LC-MS study was carried out to determine whether 16-OMC, 16-OMK or a combination of the two was responsible for the NMR signal at 3.16 ppm. Two Arabicas of assured origin (wild type, Ethiopia samples 1 and 16) and, for comparison, one robusta of assured origin (Vietnam sample 34) were selected for this study. The analysis was carried out on saponified extracts (details in the Supplementary Fig. 3).

Both cafestol and kahweol were detected in all 3 samples, as expected. Their presence was indicated by observation of peaks for mass ions ([M+H]^+^ = *m*/*z* 317, 299, 281, 147 and 133) for cafestol and (315, 297, 279, 145, 131) for kahweol (Scharnhop & Winterhalter, 2009). Note the 2 Da mass difference due to the additional carbon-carbon double bond in kahweol relative to cafestol. In addition we detected ions (331, 299, 281, 147, 133) and (329, 297, 279, 145, 131) which indicate the presence of 16-OMC and 16-OMK, respectively (Scharnhop & Winterhalter, 2009), again in both Arabicas and in the robusta coffee. These series are the same as cafestol and kahweol but with additional ion masses at 331 and 329 Da due to the extra methyl group. These two ions are therefore important for the identification of 16-OMC and 16-OMK. Extracted ion chromatograms show retention times of 35.41 min for cafestol and 37.10 min for 16-OMC (*m*/*z* = 299.201 Da), and 35.26 min for kahweol and 37.52 min for 16-OMK (*m*/*z* = 297.18 Da). The LC-MS data also revealed the presence of dehydrocafestol and dehydrokahweol in both Arabica and robusta. However these two compounds do not contribute to an NMR peak at 3.16 ppm.

In summary, an NMR peak at 3.16 ppm, detected in both Arabica and robusta ground coffees, is consistent with *both* 16-OMC and 16-OMK, based on known ^1^H spectral peaks and on 2D NMR analysis. Separately, LC-MS shows that both Arabica and robusta ground coffees contain 16-OMC and 16-OMK. Therefore, we conclude that the Arabica and robusta samples tested contain *both* 16-OMC and 16-OMK and that these compounds contribute to the 3.16 ppm ^1^H NMR marker peak. We hypothesise that all Arabicas and robustas displaying a 3.16 ppm peak contain either 16-OMC or 16-OMK or a mixture of both, with other candidate compounds very unlikely to satisfy the NMR and LC-MS evidence presented here. For the remainder of the present work we therefore assume that the ^1^H NMR 3.16 ppm peak is entirely due to 16-OMC and 16-OMK (in esterified form in both robusta and Arabica, since diterpenes are present in coffee almost exclusively in this form). Neither of these assumptions invalidate our authenticity test based on the 3.16 ppm ^1^H NMR peak area. In contrast, attempts to quantify 16-OMC on the basis of the 3.16 ppm ^1^H NMR peak area are likely to be a quantification of both 16-OMC and 16-OMK in combination, since these two compounds are indistinguishable at 3.16 ppm using ^1^H NMR at field strengths up to 600 MHz. Therefore quoted values of 16-OMC for robusta determined by integration of the 3.16 ppm peak are likely to be overestimates unless the contribution due to 16-OMK has been determined and subtracted from the total integrated area. In addition, comparisons of the level of 16-OMC determined via ^1^H NMR 3.16 ppm peak areas are likely to be systematically larger than those derived from the DIN method using only a 16-OMC chromatography peak and well-resolved chromatography.

The presence of 16-OMC in robusta is well-known. 16-OMK was first reported and quantified in robusta coffee by Kölling-Speer et al., 2001, Kölling-Speer and Speer, 2001). However the present work is the first to explicitly report the observation of the two compounds 16-OMC and 16-OMK in Arabica roast coffee. It is therefore useful to contrast the method set out in the DIN standard with our LC-MS method. The work flow is comparable: extraction, liquid chromatography and detection. However we have increased the extractable yield of 16-OMC available for detection. More importantly, we are now using a mass spectrometer as the detector, which is more sensitive than using UV detection at 220 nm as used for the DIN method. This has the added benefit of giving additional information on the species under the chromatography peaks, whereas a UV absorbance quantitation method is sensitive to the presence of an unknown compound that coelutes with the 16-OMC target.

### 3.3 The NMR marker in samples of assured origin

**Fig. 4:**
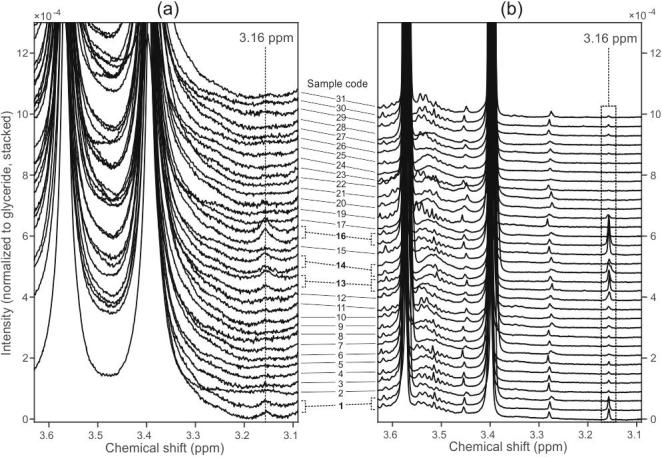
The region around the 3.16 ppm peak in spectra acquired from 30 Arabica coffees of assured origin, by (a) 60 MHz and (b) 600 MHz NMR. In both cases the spectra have been internally normalized to the glyceride peaks to facilitate side-by-side comparison on the same vertical scale.

Spectra were collected from all 30 Arabica coffees of assured origin (Supplementary Table 1, samples 1–30). The region of interest in the 60 MHz and 600 MHz spectral collections are shown in Fig. 4. The presence of the 3.16 ppm marker peak was confirmed in every sample, irrespective of genetic background and provenance. In view of these results, we propose that 16-OMC and 16-OMK is likely to be present at low levels in most if not all Arabica coffee beans, but in many previous studies had not been detected (Bonnlander et al., 2007, de Roos et al., 1997, Defernez et al., 2017, Kamm et al., 2002, Kurzrock and Speer, 2001, Pacetti et al., 2012, Speer and Kölling-Speer, 2006, Speer and Mischnick, 1989). Indeed, our own ability to detect a peak has been greatly improved by altering the extraction procedure, specifically, by using a much greater amount of coffee to carry out the extraction. In other work, 16-OMC was detected and quantified but its presence at low levels in declared “100% Arabica” coffees dismissed on the grounds that the relative integral of the 16-OMC peak areas were below a threshold (Monakhova et al., 2015). It is also pertinent to note that 16-OMC has previously been reported in other parts of the Arabica coffee plant (Speer and Kölling-Speer, 2006) and 16-OMK in leaves (Kölling-Speer & Speer, 2001).

Of particular note are four of the wild type samples (samples 1 [Bale], 13 [Konso], 14 [Jinka] and 16 [Western Harar]) which show much larger 3.16 ppm marker than the other samples. The extraction procedure and spectral acquisition at both field strengths was repeated for these samples; these technical replicates are indicated by brackets on Fig. 4. These samples are all from marginal coffee-growing areas, either from the eastern side of the Great Rift Valley (Bale, Western Harar), or from the Rift area (Jinka and Konso). However, the other Ethiopian wild type samples from east of the Rift Valley (samples 2, 3, 17) and the Rift area (sample 15 [Gidole]) do not show elevated levels of 3.16 ppm marker. The reasons for these differences are unclear. There does not seem to be a relationship with climate or altitude.

Arabicas of this type could in principle show positive for robusta contamination in a very sensitive test regime simply because they naturally give rise to a large 3.16 ppm peak. However it is important to emphasize that samples 1, 13, 14 and 16 are from low-producing, niche coffee farming areas (i.e. the non-commodity sector). If traded, they would be placed in the higher value ‘Speciality Coffee’ sector, with their provenance known to the importer or coffee buyer. Furthermore, Ethiopia only grows an extremely limited amount of this species (see Methods), with no recorded production or export of robusta (International Coffee Organization., 2017a).

**Fig. 5:**
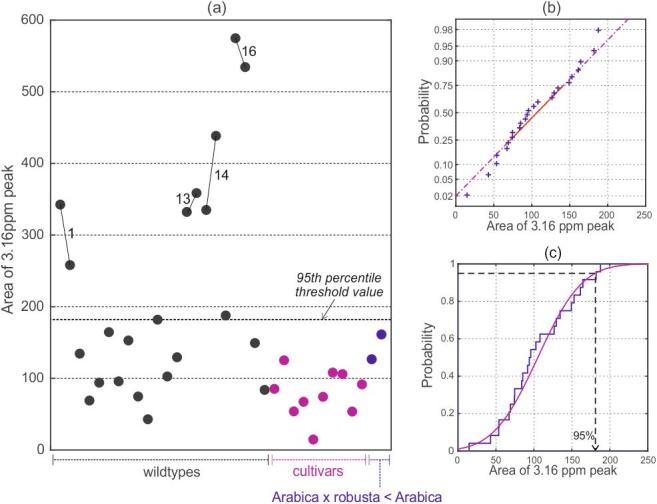
(a) The integrated 3.16 ppm peak areas in 60 MHz spectra from the assured source Arabicas. Samples 1, 13, 14 and 16 originate from atypical coffee-growing locations. Replicate measurements made on repeat extractions from these samples are indicated by joined points. (b) Normal probability plot for the data in (a) (excluding the atypical samples). (c) Empirical and fitted cumulative distribution functions for typical Arabica coffees.

The integrated area of the 3.16 ppm peak in each of the 60 MHz spectra is shown in Fig. 5(a). The technical replicates carried out on the four samples from marginal coffee-growing areas give an impression of the reproducibility of the method. The variation in peak area values may reflect the greater genetic diversity of wild type Ethiopian Arabicas in comparison to the cultivated Arabicas (Aga et al., 2005, Aga et al., 2003, Anthony et al., 2001, Lashermes et al., 1996, Montagnon and Bouharmont, 1996) originating from outside of the species indigenous range of Ethiopia and South Sudan (Davis, Gole, Baena, & Moat, 2012). It is also noteworthy that the two backcrossed Arabica × robusta < Arabica samples do not show significantly larger 3.16 ppm peaks, despite their genetic heritage.

The 600 MHz spectra were similarly analysed and the same pattern of results was found. Indeed, the correlation between the 60 MHz and 600 MHz peak area values in analogous experiments is remarkable (see Supplementary Fig. 4). At this juncture, we conclude that for the analysis of a single, isolated resonance such as the 3.16 ppm peak, there is no benefit in carrying out NMR spectroscopy at the higher field strength. The 60 MHz approach offers considerable advantages in terms of ease-of-use, as well as lower capital and maintenance costs.

### 3.4 Methodology development for authenticity testing

A consequence of finding a measurable 3.16 ppm marker peak in Arabicas is that any authenticity test has to be based upon the distribution of its naturally occurring level in authentic Arabica samples, rather than relying on it as a simple ‘present/absent’ marker compound. Fortunately, the 26 Arabica coffees from typical coffee-growing regions (samples 1–30, but excluding 1, 13, 14 and 16) form a well-behaved normal population with regards to the 3.16 ppm peak (Fig. 5(b)). This enables us to choose an upper threshold for the peak area, at an appropriate probability level, for use in a test to verify the authenticity of Arabica coffees. The value of the integrated peak area corresponding to the 95th percentile is marked on Fig. 5(c), and also on 5(a). Working at this level, we can expect 5% of authentic Arabicas to be wrongly assessed as suspicious (Type I errors).

**Fig. 6:**
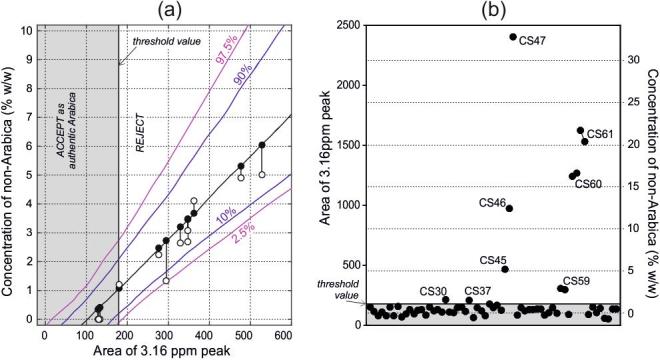
(a) The low concentration region in a calibration chart developed to estimate the concentration of adulterant (robusta or other non-Arabica) present in samples that fail to be accepted as authentic Arabicas. The calibration line (black) indicates the median of the regression lines obtained by simple linear regression onto all possible pair-wise combinations of Arabica and non-Arabicas. Percentiles are as indicated (coloured lines). The open and closed markers indicate the actual and predicted concentrations for the mixture series, with the error indicated by vertical lines. (b) The integrated 3.16 ppm peak areas for the 60 surveillance samples. The right-hand vertical axis is an equivalent concentration scale obtained from the calibration line in (a).

In contrast, the incidence of Type II errors (incorrectly accepting suspicious samples as authentic) is harder to estimate as it depends on the detection limits of *any, unidentified* non-Arabica in *any* declared Arabica sample. To illustrate this issue, simple linear regression was used to construct a universal calibration for the quantity of non-Arabica in Arabica coffee, using the assured origin samples (the 26 typical Arabicas and 10 non-Arabicas, see Supplementary Table 1). This calibration was then used to estimate the level of adulteration in a range of test samples, namely the mixtures prepared in-house. The chart and details of the calibration construction are given in Supplementary Fig. 5, and an expansion of the low-concentration region in Fig. 6(a).

The threshold value calculated in the paragraph above is marked on the horizontal axis. For samples with a peak area below this value, the null hypothesis (that the sample is an authentic Arabica) is accepted. For peak areas above this value, the sample is considered suspicious, and the calibration can be used to estimate its non-Arabica content.

The calibration line is marked on the chart, along with associated confidence intervals. The main source of uncertainty in the calibration is the variation in the magnitude of the 3.16 ppm marker of non-Arabica coffees. This can be seen from the peak areas for these samples, which are plotted in the full-range chart shown in Supplementary Fig. 5. This is consistent with the literature, which suggests that the concentration of 16-OMC in robusta coffees varies considerably (de Roos et al., 1997, Finotello et al., 2017, Kurzrock and Speer, 2001, Mori et al., 2016, Pettitt, 1987, Scharnhop and Winterhalter, 2009, Schievano et al., 2014). By way of example, using NMR Finotello et al. quote the level of 16-OMC as ranging from 1204 to 2236 mg/kg for a selection of roasted robustas from across the world (Finotello et al., 2017). Using the traditional DIN HPLC-based method Kurzrock and Speer quote 600–1800 mg/kg (Kurzrock & Speer, 2001). This variance notwithstanding, comparison of the median peak areas for Arabica and robusta coffees suggests that the combined 16-OMC and 16-OMK content of a typical Arabica is approximately 1.5% that of a typical robusta.

To test the calibration, the peak areas from the mixture series were plotted on the calibration chart against their known concentrations (empty circles), Fig. 6. Applying the threshold to the peak areas, all three “0% w/w robusta” extracts are accepted as authentic Arabicas. Further, all the remaining mixture samples are flagged as suspicious, including that with the lowest concentration of robusta (1.2% w/w).

The concentrations predicted by the calibration for these samples are marked by filled circles, and the errors in prediction shown as vertical lines. A table detailing the outcomes of the test samples (all in-house mixtures) is included in Supplementary Fig. 5. The confidence intervals for the predictions illustrate the unavoidable uncertainty that arises from the adulterant non-Arabicas being of unknown (and unknowable) chemical composition.

More generally, the calibration chart suggests that Arabicas adulterated with robusta at the 1% w/w level will be flagged as suspicious in around half of all cases. At 2% w/w robusta, this rises to 90%; and at 3% w/w, it is unlikely that an adulterated sample will pass undetected.

To ease comparison with the existing literature, quantitative NMR analysis, using 600 MHz spectroscopy only, was carried out by spiking a sample of Arabica beans of assured origin with freeform 16-OMC. The region of interest in the series of samples is shown in Supplementary Fig. 6(a), along with the calibration curve (Supplementary Fig. 6(b)) for the integrated 3.16 ppm peak area versus added 16-OMC content. From this, the concentration of combined 16-OMC and 16-OMK in the unspiked Arabica is estimated to be at least 10 mg/kg (±4 mg/kg) (recognizing that the extraction efficiency is likely less than 100%). Details of the calculations are given in the figure. Note that the sample selected for this analysis (sample 12) was a typical wildtype Arabica, with a mid-range 3.16 ppm peak area as assessed in the section above.

A plot showing the signal-to-noise ratio (SNR) for the integrated peak in each case versus the estimated 16-OMC plus 16-OMK content is shown (for the low level spiked samples only, in the interests of clarity) in Supplementary Fig. 6(c). From this, we determined our LoD (SNR = 3 of combined 16-OMC and 16-OMK) in roast coffee beans to be 1 mg/kg, and our LoQ (SNR = 10) to be 4 mg/kg. These limits are lower by a factor of 5 than those reported in (Schievano et al., 2014), for instance, who quote 5 mg/kg and 20 mg/kg for the LOD and LOQ respectively. They are also consistent with the estimates of the errors involved in the calculation of the combined 16-OMC and 16-OMK content of the sample. Near identical values of the LOD and LOQ (1 and 4 mg/kg respectively) were obtained by the alternative method using Arabica/robusta mixtures and an internal concentration standard, DMF (Supplementary Fig. 7). By way of comparison, the DIN 10779 method states it was tested for a mass fraction of 50 mg to 300 mg/kg of 16-OMC in ground roast coffee. A limit of detection of 10 mg/kg was stated using DIN 10779 by Kölling-Speer et al. (2001).

### 3.5 Surveillance study

Finally, a surveillance study was conducted to look for instances of fraud in premium “100% Arabica” ground roast coffees obtained from the retail and catering sectors. The method outlined here can detect of the order of 1% w/w robusta in Arabica, so if substitution has occurred in a sample at this level or above, it will likely be discovered. The probability of finding some instances of fraud in a surveillance exercise then depends on the number of coffees examined and the prevalence of fraud in the sector. It can be calculated using the binomial sampling theorem for different survey sizes and prevalences (Supplementary Fig. 8(a)).

Reported incidents of fraud involving substitution of Arabica with robusta are few (Interpol., 2015). It is probable that many cases go unrecognized (Toci et al., 2016), so the prevalence of fraud is *a priori* unknown. This being so, we elected to use a survey size of 60, for which the probability of finding at least one suspicious sample is at least 99% if the sector-wide prevalence is >7.5% (and respectively 95% and 90% for prevalences of >5% and >4%). We regard this as a reasonable compromise between the effort required to source large numbers of samples from a worldwide industry, and the chance of failing to find any suspicious samples should the prevalence of fraud in the sector be very low.

Each of the 60 survey samples were prepared following the protocol described above. The integrated area of the 3.16 ppm peak in the 60 MHz spectra from each sample is shown in Fig. 6(b). It is immediately obvious that there are several suspicious samples. Eight samples have 3.16 ppm peak areas above the threshold value and are rejected as authentic Arabicas (p < .05). Of these, 2 are only slightly above the threshold value. The other 6, however, have estimated non-Arabica contents ranging from 3 to 33% w/w. This can be seen by reading values from the right-hand vertical axis, which indicates an equivalent concentration scale obtained using the established calibration line.

These values are in excess of what could reasonably be claimed as adventitious contamination. We suggest this is highly indicative of fraudulent substitution, or at the very least, unacceptably poor quality control. For three of these, sufficient sample was available to carry out repeat extractions. The outcomes for these are also shown on the plot and detailed in full in Supplementary Table 3, again illustrating the reproducibility of the method.

The prevalence of fraud in the sector can be estimated from the survey size and the number of cases discovered. Giving the two marginal cases the benefit of the doubt, we can say that 6 instances of fraud have been found. This leads to an estimate of the sector-wide prevalence of between 5% and 20% (95% confidence interval; see Supplementary Fig. 8(b) and (c)).

## 4 Conclusions

We have described an improved preparation procedure for extracting the lipophilic phase from ground roast coffees, which produces a more concentrated extract and a resultant increase in NMR SNR. As a consequence, we have been able to detect a peak at 3.16 ppm not only in robusta, as has been described previously by several groups, but also in Arabica coffees. This is a surprising and wholly unexpected result. The Arabica peak is a small but well-resolved resonance at both 60 MHz and 600 MHz field. Two-dimensional NMR experiments (HSQC and HMBC) showed this could arise from 16-OMC or 16-OMK in both Arabica and robusta, and that it cannot arise from the other major coffee diterpenes. LC-MS carried out on Arabica samples (wild type, Ethiopian, authenticity assured by in-situ collection), unequivocally showed the presence of both 16-OMC and 16-OMK. A study of the levels of these two compounds in the Arabica population is beyond the scope of this manuscript, though may be the object of further work.

The 3.16 ppm peak was detected in all but one Arabica coffees of assured origin that we examined (17 wild type Ethiopian, 1 wild-type Columbian, 12 cultivars from various coffee producing countries). This is a significant finding, as Arabica coffees had been presumed to contain no 16-OMC (or 16-OMK), in contrast to other coffee species. Indeed, this presumption underpins the recognized DIN method (DIN 10779, 2011) for authenticating Arabica coffee products. Beyond this being of interest in terms of biology, one also should examine the practical implications for authenticity testing. These depend entirely on the detection limit of the approach used: in the present work, we were not able to use the existence of the 3.16 ppm peak as a simple presence/absence test for robusta (see below), as we and others had done in previous work (note this peak was previously believed to only arise from 16-OMC in robusta, whereas it likely arises from both 16-OMC and 16-OMK). However, the usage of other methods may not need to be affected if their detection limit for 16-OMC is above the levels observed in authentic Arabicas, as appears to be the case for the DIN method.

A normal range for the amount of combined esterified 16-OMC and 16-OMK, as expressed by the 3.16 ppm peak area, was established for our collection of fully authenticated Arabica wild types and cultivars (details above and in Supplementary Table 1). From comparison with the peak areas measured in other coffee species, we estimate that a typical Arabica coffee contains of the order of 1–2% the level of combined esterified 16-OMC and 16-OMK of a typical robusta. This has an important corollary: it is not possible to test for robusta adulteration in Arabica coffee below the level of approximately 1% using the 16-OMC/16-OMK marker, since it is present in pure Arabica at a level commensurate with 1% robusta addition. Neither is it possible to express a single, exact detection limit for robusta in Arabica, since the levels of these compounds vary across different robustas and Arabicas.

Even though 16-OMC and 16-OMK occur naturally in Arabicas, the 3.16 ppm peak area can be used as a reasonable proxy for the robusta content in mixture samples by defining a threshold marking the upper limit of the normal Arabica range. Simple linear regressions relating the area of this peak to the quantity of robusta in known mixture samples demonstrated excellent linearity and precision at both field strengths.

Further, the correlation between the outcomes at 60 and 600 MHz was very high, from which we can infer that the source of error in the separate regressions must derive from the sample preparation (normal uncertainties associated with mass and liquid measurement) rather than the spectroscopy. We conclude that for quantitation of a single, well-resolved spectral peak, there is no advantage to using the higher field strength. Due to the nature of the instrument design, a somewhat larger volume of sample is examined in the low-field spectrometer. Along with the experimental and acquisition parameters used, this has enabled us to achieve a quantitative precision at 60 MHz comparable to that at 600 MHz. The interest in using the lower field strength stems from the advantages in affordability, robustness and ease-of-use of the 60 MHz instrument. Commercially relevant sensitivity makes industry-sited routine screening a reality for the first time.

Finally, the improved procedure was used to prepare extracts from retail samples of “100% Arabica” coffees sourced worldwide. 60 MHz and 600 MHz spectroscopy detected the 3.16 ppm peak in all 60 samples analysed, of which 52 had peak areas consistent with the naturally occurring range in authentic Arabicas. Two samples were just above the threshold value, but the remaining six display a 3.16 ppm marker peak strength strongly suggestive of adulteration with another coffee species. From standard sampling theory, we estimate that the sector-wide prevalence of fraud is in the range 5–20% of “100% Arabica” ground roast coffee products.

## Author contributions

The manuscript was written through contributions from all authors with input as follows: experimental work: YG, NB, GLG, IJC, MP, HG; data analysis: EKK, MD, GLG, IJC; planning and oversight: MD, ADW, DW, AD, EKK; writing up: EKK, MD, AW, YG, GLG, AD, IJC.

## Conflicts of interest

There are no conflicts of interest associated with this work.
