# Supplementary material for: 16-O-methylcafestol is present in ground roast Arabica coffees: Implications for authenticity testing
Source: Food Chem. 2018 May 15;248:52–60. doi: 10.1016/j.foodchem.2017.12.034 (PMC5774150; doi:10.1016/j.foodchem.2017.12.034)
Supplement: Supplementary data [file mmc1.pdf]

Supplementary Table 1: Details of Arabica and "non-Arabica" coffees of assured origin (RBG, Kew), together with 16-OMC + 16-OMK content measured by high-field NMR.

Sample Code	Species/hybrid	Category	Cultivar (where applicable)	Country of origin	Location (where known)	Measured16-OMC + 16-OMK content from 3.16ppm peak (mg/kg)
Arabica coffees, wild type						
1	Arabica	wild type		Ethiopia	Bale Mts.	39
2	Arabica	wild type		Ethiopia	Yirgacheffe (town centre)	9
3	Arabica	wild type		Ethiopia	Yirgacheffe (generic)	4
4	Arabica	wild type		Ethiopia	Yayu (Geri)	6
5	Arabica	wild type	Limu	Ethiopia	Jimma/Limu	8
6	Arabica	wild type		Ethiopia	Wembera (low)	7
7	Arabica	wild type		Ethiopia	Wembera (high)	6
8	Arabica	wild type		Ethiopia	Bahir Dar (Zeghie)	13
9	Arabica	wild type		Ethiopia	Ashi	3
10	Arabica	wild type	Lekemte	Ethiopia	Gimbi	8
11	Arabica	wild type		Ethiopia	Bonga (washed)	4
12	Arabica	wild type		Ethiopia	Bonga (natural)	14
13	Arabica	wild type		Ethiopia	Konso	40
14	Arabica	wild type		Ethiopia	Jinka	49
15	Arabica	wild type		Ethiopia	Gidole	16
16	Arabica	wild type		Ethiopia	Western Harar (Gololocha)	100
17	Arabica	wild type		Ethiopia	Guji	12
18	Arabica	wild type/cultivar	Geisha	Colombia		2
Arabica coffees, cultivar						
19	Arabica	cultivar	Typica	Colombia		19
20	Arabica	cultivar	Bourbon	Colombia		10
21	Arabica	cultivar	Mundo Nuovo	Brazil		2
22	Arabica	cultivar	Castillo	Colombia		3
23	Arabica	cultivar	Bourbon Pointu	La Reunion		1
24	Arabica	cultivar	Laurina	Brazil		0
25	Arabica	cultivar		Uganda	Mt. Elgon	1
26	Arabica	cultivar	Caturra	Colombia		0
27	Arabica	cultivar	Caturra	Costa Rica		5
28	Arabica	cultivar	SL 28/SL 34	Kenya		3
29	Arabica x robusta < Arabica	cultivar (hybrid)	Batian	Kenya		6
30	Arabica x robusta < Arabica	cultivar (hybrid)	Ruiru 11	Kenya		4
Non-arabica coffees						
31	Arabica x robusta	cultivar (hybrid)	Arabusta	Kenya		1422
32	canephora		S. 274	India		2143
33	canephora			Indonesia		2281
34	canephora			Vietnam		1873
35	canephora			Brazil		1292
36	canephora			Rwanda		1563
37	canephora			India		2132
38	congesis x robusta	hybrid		India		2132
39	liberica			Uganda		1189
40	liberica			Uganda		1139

Supplementary Table 2: Details of retail coffees used in the surveillance exercise, together with 16-OMC + 16-OMK content measured by high-field NMR.

Sample Code	Country of Purchase	Country of Coffee Origin as stated on label	Measured 16-OMC + 16-OMK content from 3.16ppm peak (mg/kg)
CS01	Poland	Not specified	3
CS02	Poland	Not specified	8
CS03	Poland	Brazil	8
CS11	France	Not specified	4
CS12	France	Not specified	12
CS13	France	Not specified	8
CS14	France	Ethiopia	6
CS18	The Netherlands	Not specified	3
CS19	UK	Java	13
CS20	UK	Kenya	0
CS21	UK	Colombia	0
CS22	UK	Colombia	5
CS23	UK	Kenya	1
CS25	UK	Africa, Indonesia, Latin America	0
CS26	UK	Not specified	1
CS27	UK	Colombia	1
CS28	UK	Colombia	10
CS29	UK	Kenya	15
CS30	UK	Not specified	16
CS31	UK	Guatemala	4
CS32	UK	Not specified	9
CS33	UK	Not specified	9
CS34	UK	Not specified	9
CS36	Italy	Not specified	5
CS37	France	Not specified	13
CS39	France	Not specified	10
CS40	Germany	Not specified	5
CS41	France	Not specified	4
CS42	Italy	Not specified	4
CS43	Spain	Colombia	25
CS44	Italy	Nicaragua	21
CS45	USA	Not specified	76
CS46	USA	Not specified	208
CS47	USA	Not specified	528
CS48	USA	Not specified	5
CS49	USA	Not specified	12
CS50	The Netherlands	Not specified	11
CS51	The Netherlands	Not specified	4
CS53	USA	Not specified	3
CS54	USA	Mexico	22
CS55	USA	Mexico	4
CS56	USA	Not specified	5
CS57	Australia	Not specified	4
CS58	Italy	Not specified	9
CS59	Italy	Not specified	41
CS60	Estonia	Not specified	212
CS61	UK	Mexico	351
CS62	UK	Costa Rica	4
CS63	UK	Brazil	0
CS64	UK	Brazil	4
CS65	UK	Ethiopia	13
CS66	Italy	Costa Rica	6
CS67	UK	Brazil, Nicaragua, Honduras	6
CS68	UK	Brazil, Nicaragua, Honduras	2
DH1	UK	Ethiopia	5

DH2	UK	Brazil	6
DH3	UK	Guatemala	1
DH4	UK	Colombia	3
DH5	UK	Kenya	3
DH6	UK	Not specified	0

Supplementary Table 3: Detailed outcomes for the surveillance samples flagged as suspicious by high-field NMR

Sample code	p-value (H0: sample is authentic Arabica)	Estimated concentration of robusta (% w/w)	95% confidence interval for the concentration estimate (% w/w)
CS30	0.01	1.6	0.7 - 2.9
CS37	0.01	1.5	0.6 - 2.9
CS45	1×10^{-16}	5.2	3.1 - 9.1
CS46	$< 1 \times 10^{-16}$	12.4	8.1 - 21.6
CS47	$< 1 \times 10^{-16}$	32.8	22.0 - 56.5
CS59	1×10^{-6}	2.9	1.6 - 5.2
CS59 (replicate extract)	1×10^{-6}	2.8	1.5 - 5.0
CS60	$< 1 \times 10^{-16}$	16.2	10.7 - 28.0
CS60 (replicate extract)	$< 1 \times 10^{-16}$	16.6	10.9 - 28.8
CS61	$< 1 \times 10^{-16}$	21.7	14.4 - 37.5
CS61 (replicate extract)	$< 1 \times 10^{-16}$	20.4	13.5 - 35.2

Supplementary Figure 1

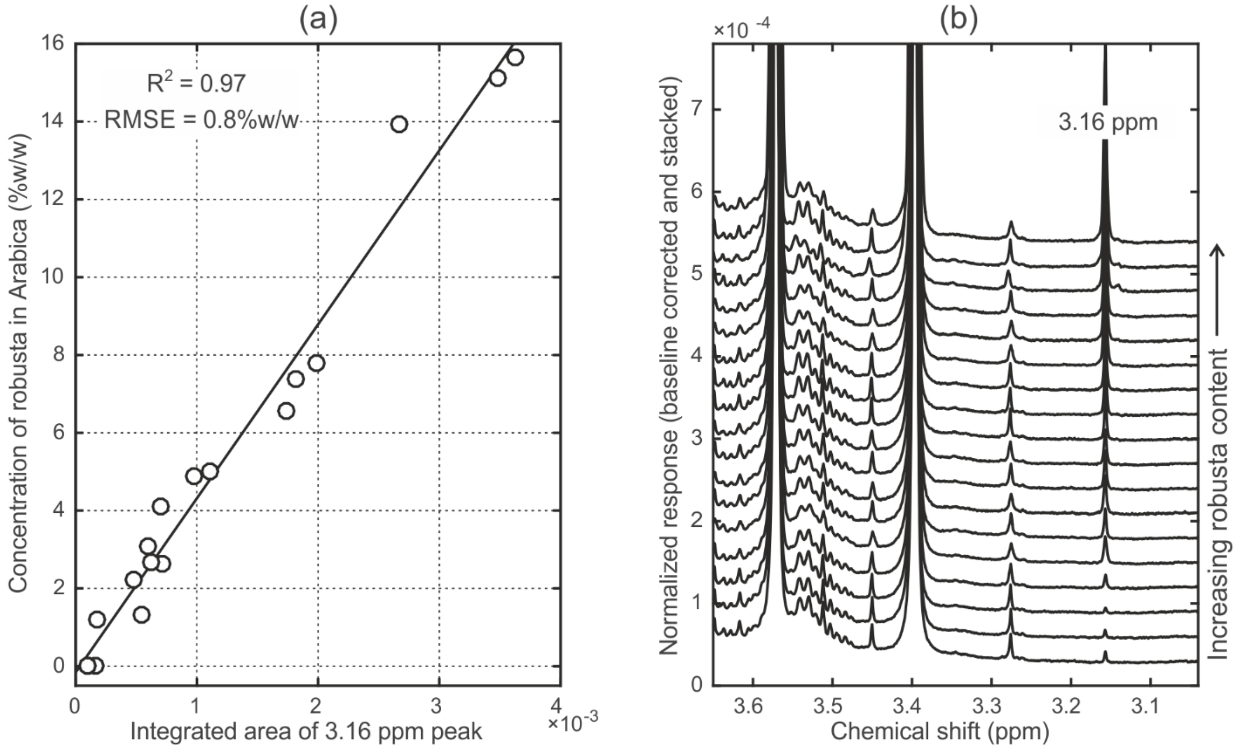

(a) Concentration of robusta in the mixture series versus integrated area of the 3.16 ppm peak in 600 MHz spectra, and the associated simple linear regression line. (b) The spectra used to obtain the calibration, shown as a stacked plot for clarity.

Supplementary Figure 2

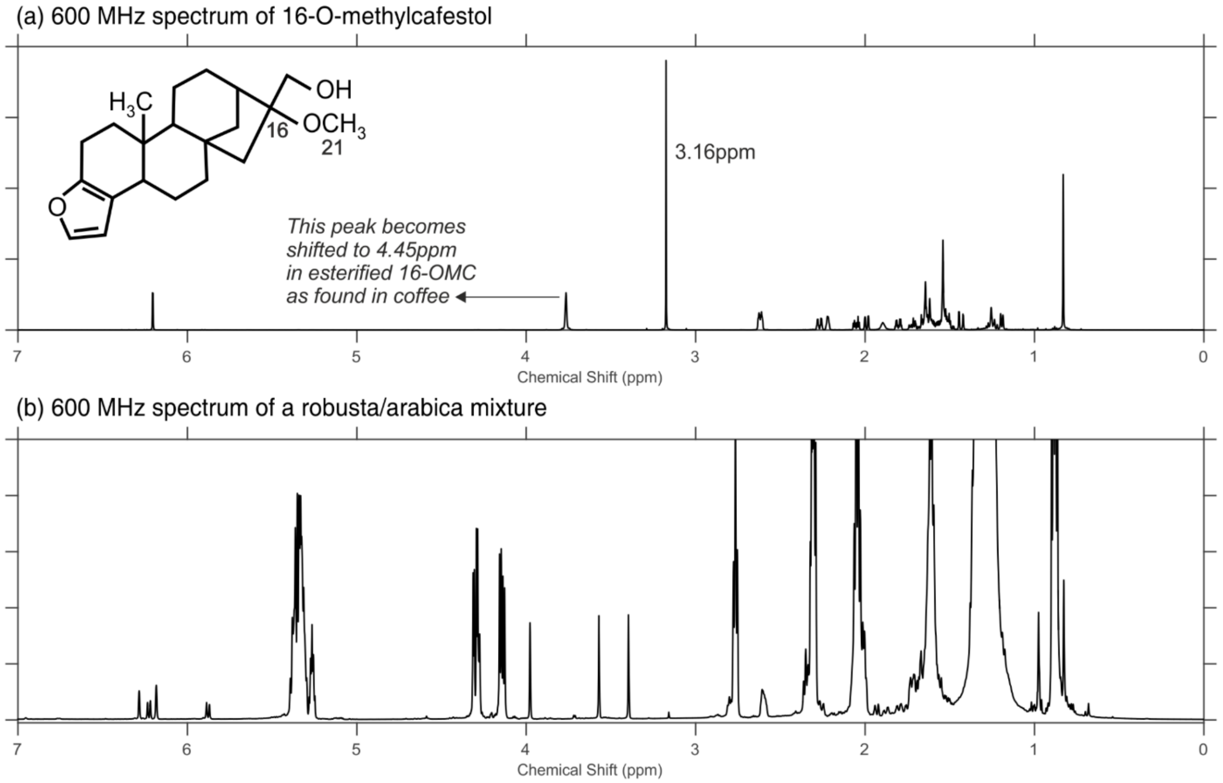

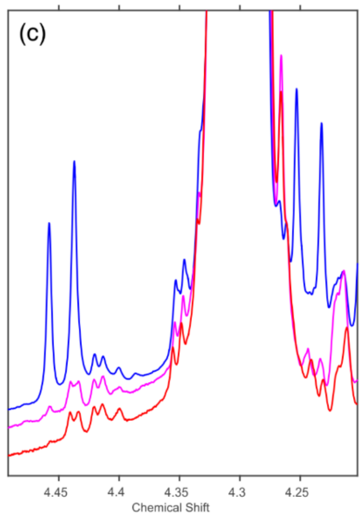

(a) 600MHz NMR spectrum of 16-OMC (analytical standard purchased from Sigma-Aldrich) in chloroform. The chemical structure of the compound is also illustrated. The C16 and 21 carbon positions are marked, as these are referred to in the main body of the manuscript. The 3.16ppm peak is the largest feature; it is also the only signal that remains both isolated and insignificantly shifted in the esterified form of the compound as found in coffee.

(b) 600MHz NMR spectrum of an extract prepared from 20 %w/w robusta, 80 %w/w Arabica mixture. The 3.16ppm peak can just be distinguished on this scale.

(c) Zoom of the region 4.2 - 4.5ppm in extracts from a robusta (blue trace) and two Arabica coffees. This is the region into which the peak at 3.77ppm is shifted in esterified 16-OMC, the form that predominates in coffee.

Supplementary Figure 3

Instrument settings for UPLC-MS analysis

Two Arabica (1, 16) and one Robusta (34) samples were selected for analysis.

The UPLC-MS analysis was carried out using a Waters Acquity UPLC interfaced to Waters Synapt G2-Si QTOF instrument.
Column: Waters HSS T3 C18 100mm x 2.1mm x 1.7µ
Inj = 5µl
Flow = 400 µl/min
A = Water + 0.1% FA
B= Acetonitrile + 0.1% FA
MS
POS MSe mode (dual collision energy)
50-1200Da
0.3 scan/sec
Gradient:

Time	%A	%B
0	95	5
1	95	5
5	90	10
30	75	25
40	5	95
41	5	95
41.1	95	5
46	95	5

Polarity	ES+
Capillary (kV)	2.5000
Source Temperature (°C)	140
Sampling Cone	28.0000
Source Offset	80.0000
Source Gas Flow (mL/min)	0.00
Desolvation Temperature (°C)	400
Cone Gas Flow (L/Hr)	10.0
Desolvation Gas Flow (L/Hr)	900.0
Nebuliser Gas Flow (Bar)	4.0
LM Resolution	4.9
HM Resolution	15.0
Aperture 1	0.0
Pre-filter	2.0
Ion Energy	1.0
Manual Trap Collision Energy	FALSE
Trap Collision Energy	4.0
Manual Transfer Collision Energy	FALSE
Transfer Collision Energy	2.0
Manual Gas Control	FALSE
Trap Gas Flow (mL/min)	2.00
HeliumCellGasFlow	180.00
IMS Gas Flow (mL/min)	90.00

Supplementary Figure 3 (a)

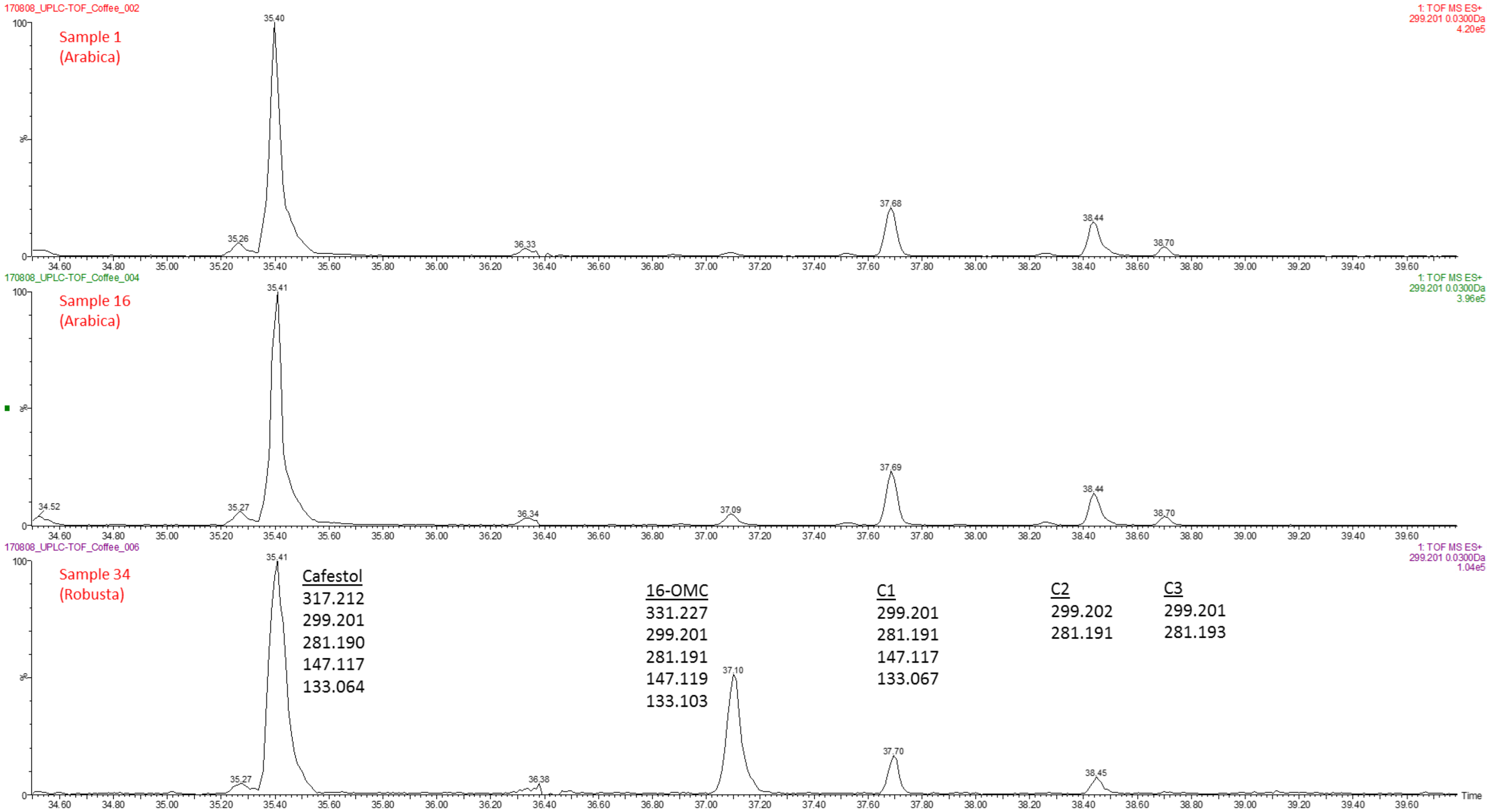

EIC of m/z 299.2 for the three coffee samples showing m/z of molecular ion and fragments: Cafestol R_t= 35.4, (theor. M+H$^+$=317.2111); 16-OMC R_t= 37.1, (theor. M+H$^+$=331.2267); C1, C2, C3 are additional cafestol related compounds (e.g. Dehydrocafestol).

Supplementary Figure 3 (b)

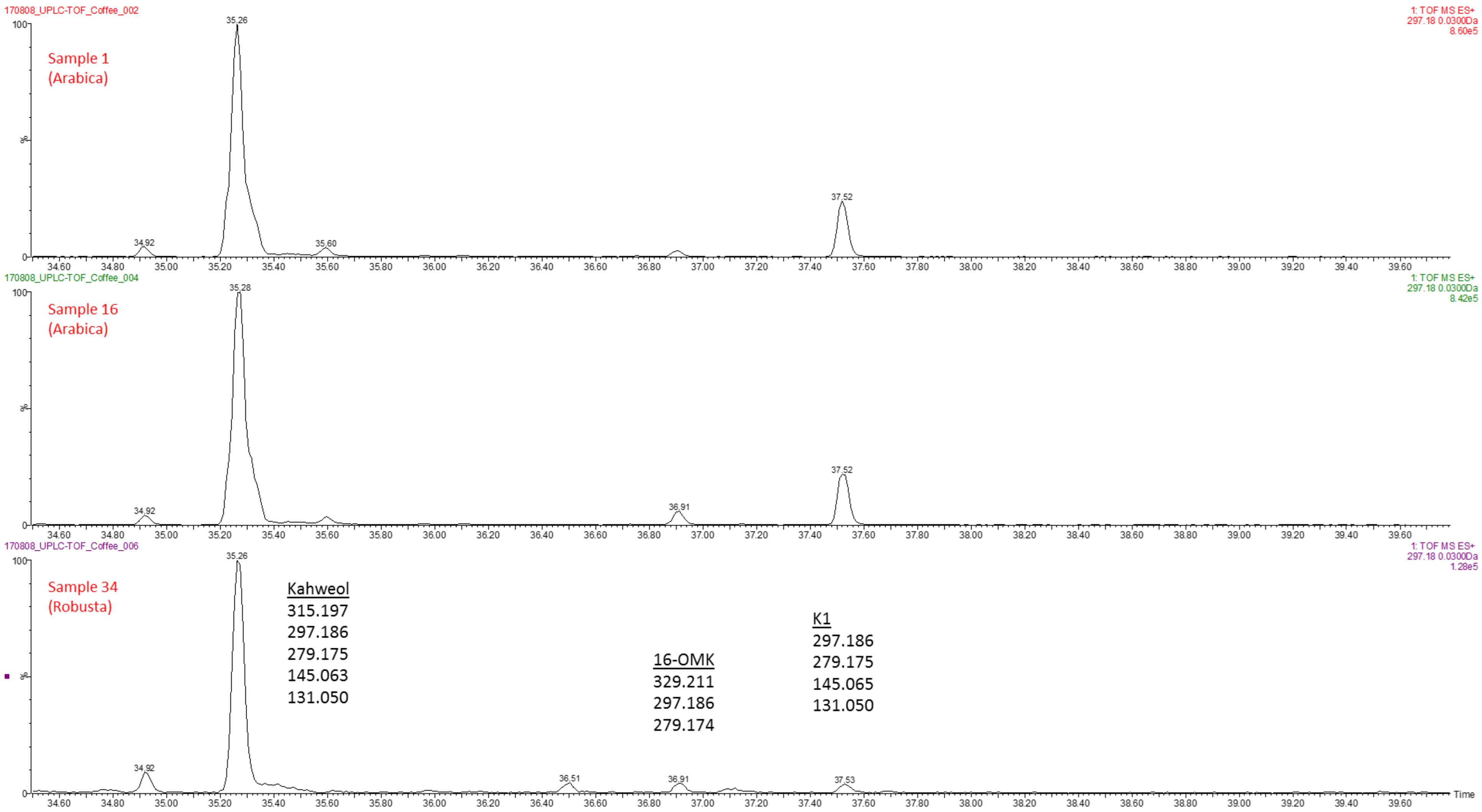

EIC of m/z 297.2 for the three coffee samples showing m/z of molecular ion and fragments: Kahweol R_t= 35.26, (theor. M+H$^+$=315.1955); 16-OMK R_t= 36.9, (theor. M+H$^+$=329.2111); K1 is an additional kahweol related compound (e.g. Dehydrokahweol).

Supplementary Figure 3 (c)

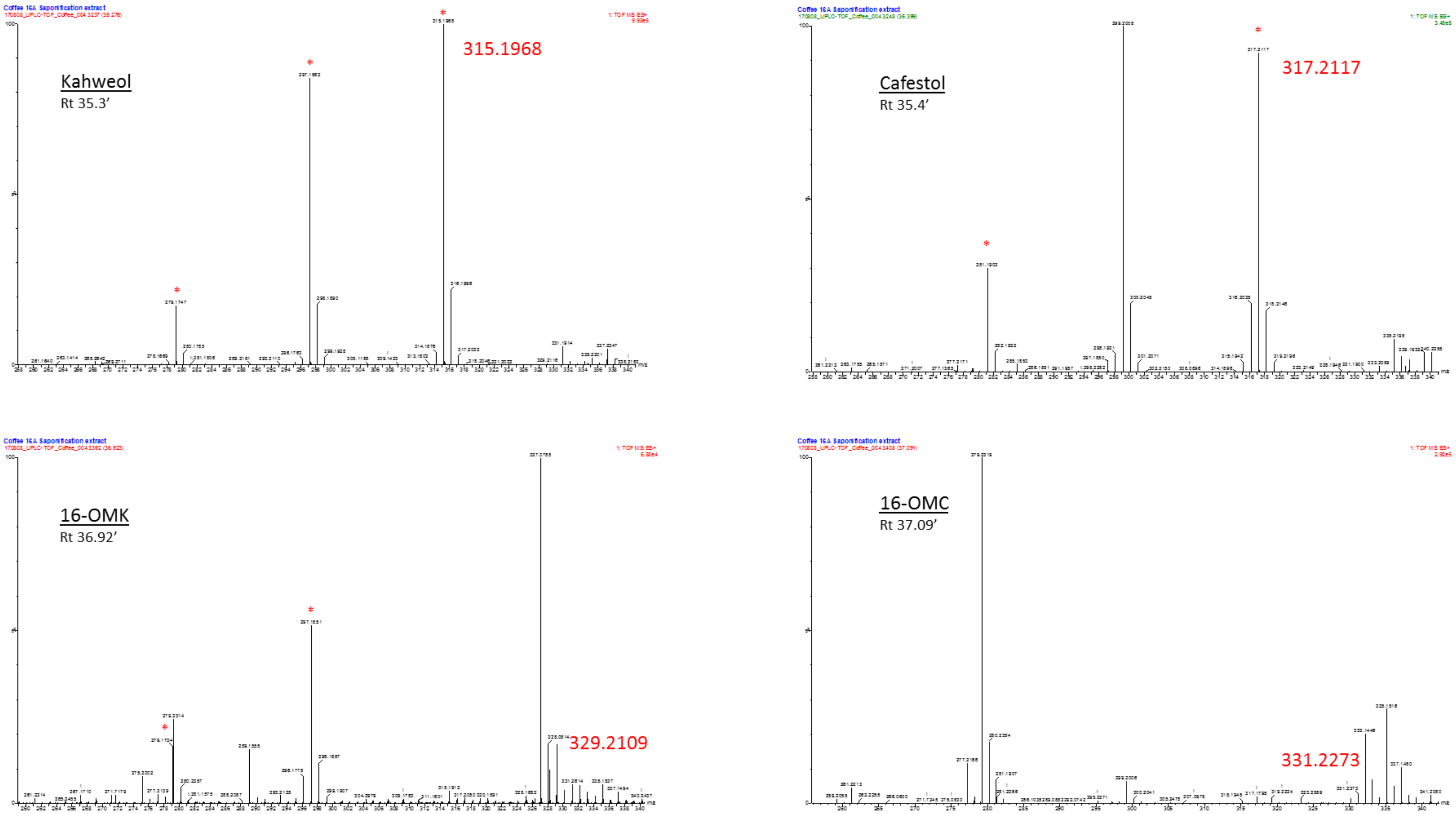

Mass spectra of compounds of interest in sample 16 (Arabica) highlighting molecular ions and key fragments.

Supplementary Figure 4

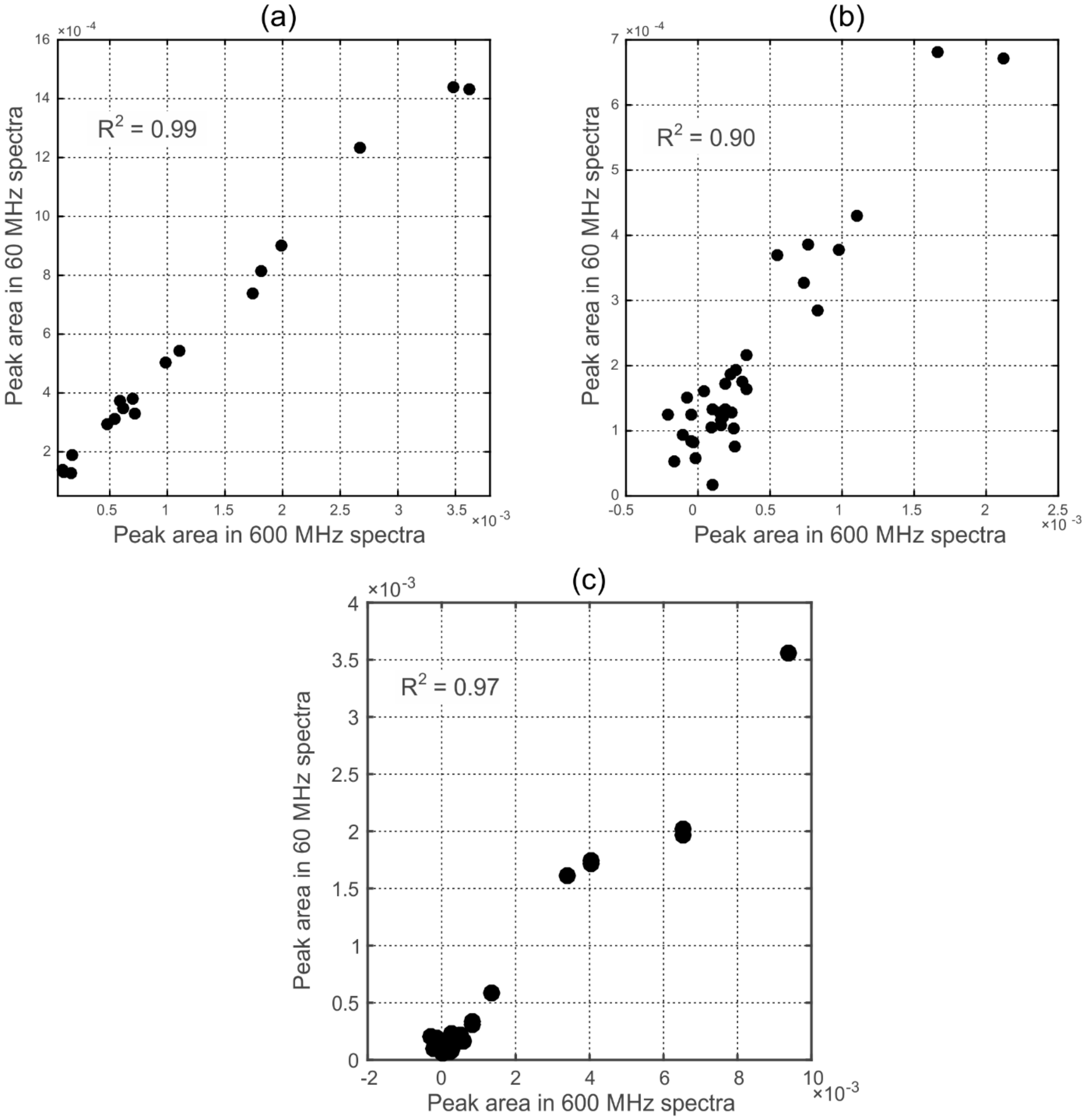

Plots of the 3.16 ppm peak area in 60 MHz versus 600 MHz spectra (glyceride-normalized), for (a) the 18 mixture series extracts; (b) extracts from the Arabicas of assured origin; and (c) for extracts from the 60 survey samples.

Supplementary Figure 5

(a)

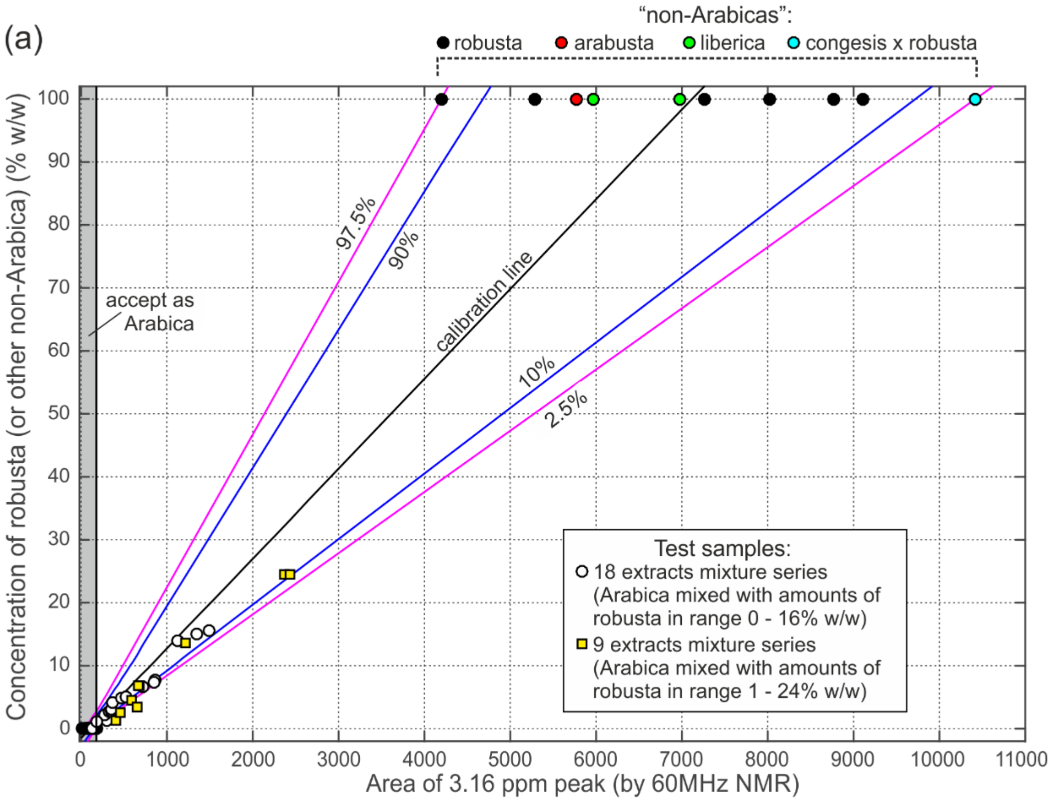

(b)

Test sample:	p-value (H0: accept as authentic arabica)	Known Robusta Concentration (% w/w)	Predicted Robusta Concentration (% w/w)	95% confidence interval for prediction (%w/w)	Error (Predicted minus Known) (% w/w)
18-Mixture series	0.30	0.00	*N/A - accept as Arabica*		
18-Mixture series	0.26	0.00	*N/A - accept as Arabica*		
18-Mixture series	0.29	0.00	*N/A - accept as Arabica*		
18-Mixture series	0.05	1.21	1.1	0.3 - 2.1	-0.1
18-Mixture series	1×10^{-6}	1.33	2.7	1.5 - 4.9	1.4
18-Mixture series	1×10^{-5}	2.24	2.5	1.3 - 4.5	0.2
18-Mixture series	1×10^{-7}	2.65	3.2	1.8 - 5.7	0.6
18-Mixture series	1×10^{-8}	2.68	3.5	2.0 - 6.2	0.8
18-Mixture series	1×10^{-8}	3.08	3.5	2.0 - 6.2	0.4
18-Mixture series	1×10^{-9}	4.10	3.7	2.1 - 6.6	-0.4
18-Mixture series	$<1 \times 10^{-16}$	4.90	5.3	3.2 - 9.4	0.4
18-Mixture series	$<1 \times 10^{-16}$	5.00	6.0	3.7 - 10.6	1.0
18-Mixture series	$<1 \times 10^{-16}$	6.57	8.8	5.6 - 15.3	2.2
18-Mixture series	$<1 \times 10^{-16}$	7.40	10.6	6.8 - 18.3	3.2
18-Mixture series	$<1 \times 10^{-16}$	7.81	10.8	7.0 - 18.7	3.0
18-Mixture series	$<1 \times 10^{-16}$	13.95	14.6	9.6 - 25.4	0.7
18-Mixture series	$<1 \times 10^{-16}$	15.12	17.7	11.7 - 30.7	2.6
18-Mixture series	$<1 \times 10^{-16}$	15.64	19.8	13.1 - 34.2	4.1
9-Mixture series	1×10^{-12}	1.22	4.4	2.6 - 7.7	3.2
9-Mixture series	1×10^{-16}	2.53	5.1	3.1 - 8.9	2.6
9-Mixture series	$<1 \times 10^{-16}$	3.36	7.9	5.0 - 13.7	4.5
9-Mixture series	$<1 \times 10^{-16}$	4.44	6.9	4.3 - 12.1	2.5
9-Mixture series	$<1 \times 10^{-16}$	6.85	8.2	5.2 - 14.2	1.3
9-Mixture series	$<1 \times 10^{-16}$	6.85	8.1	5.1 - 14.1	1.2
9-Mixture series	$<1 \times 10^{-16}$	13.65	15.9	10.4 - 27.5	2.2
9-Mixture series	$<1 \times 10^{-16}$	24.41	32.2	21.6 - 55.6	7.8
9-Mixture series	$<1 \times 10^{-16}$	24.41	33.1	22.2 - 57.1	8.7

(a) Calibration chart developed from 26 different Arabica coffees and 10 different "non-Arabicas" (robusta and other coffea species, as indicated). The "calibration line" indicates the median of the regression lines obtained by simple linear regression onto all possible pair-wise combinations of Arabica and non-Arabicas. Various percentiles are also indicated, which can be used to estimate a confidence interval for predicted concentrations. This approach exploits the excellent linearity of NMR peak areas as a function of concentration, as demonstrated in figure 2. Also marked on the chart are the known concentrations versus peak areas for 27 "test" samples (the two mixture series).
(b) The predicted % w/w values for these samples, along with the error in prediction and an estimated confidence interval. Subsequent quantitative analysis using high-field NMR estimated the 16-OMC/K content of the robusta beans used in the series to be 1288 mg/kg, a typical value for the species.

Supplementary Figure 6

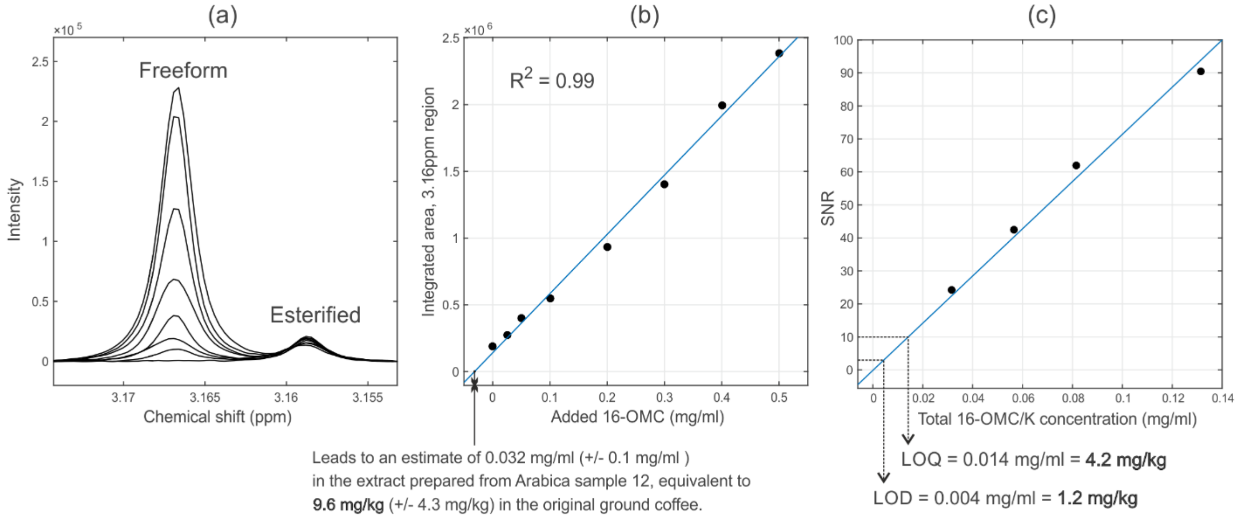

	Mass of coffee (g)	Volume of chloroform used in extraction (ml)	Volume of lipophilic extract recovered (ml)	Effective initial mass of coffee (g)	Mass of 16-OMC in 0.8ml redissolved sample (estimated from spike series calibration) (mg)	Estimated 16-OMC/K content of coffee (g/kg)
	10.00	30.0	8.0	2.67	0.026	9.6
Estimated uncertainty:	+/- 0.01	+/- 0.1	+/- 0.5	+/- 0.2	+/- 0.01	+/- 4.3
% uncertainty:	<1%	<1%	~6%	~7%	~38%	45%

Quantitative 600MHz NMR estimation of the 16-OMC/K content of Arabica sample 12, and of the LOQ and LOD, using a spiked sample series approach, where the spike is of 16-OMC analytical standard.

(a) The region of interest around 3.16ppm, clearly showing the peak from the analytical standard (freeform) and the native compound in coffee (esterified). This chemical shift difference is consistent with previously reported literature.

(b) The integrated area of the combined 3.16ppm (freeform and esterified) signals versus the known amount of added 16-OMC in the spiked solvent. The intercept of the regression line provides the estimate of the amount of 16-OMC/K in the coffee (recognising that the 3.16ppm peak may contain contributions from both 16-OMC and 16-OMK). Calculations and uncertainties used in this estimate are detailed in the table.

(c) Plot of the signal-to-noise ratio versus the total 16-OMC/K content for the same series of samples, leading to values for the limit of quantitation (LOQ) and limit of detection (LOD).

Supplementary Figure 7

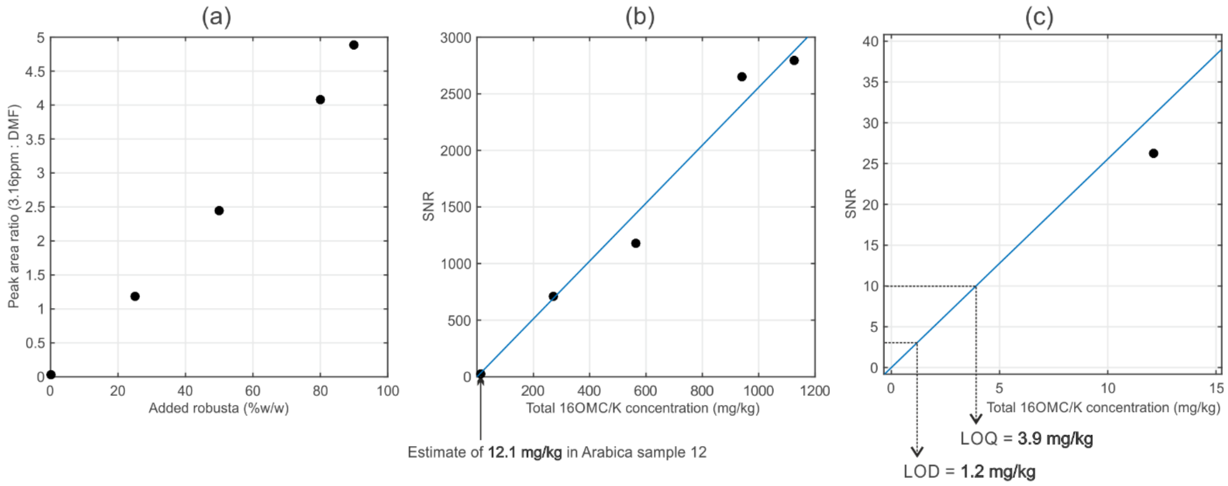

Quantitative 600MHz NMR estimation of the 16-OMC/K content of Arabica sample 12, and of the LOQ and LOD, using an alternative spiked sample series approach, where the spikes are of a robusta coffee and an NMR standard (DMF).

(a) The ratio of the 16-OMC/K to DMF peak areas versus the percentage added robusta. Using the molecular weight of 16-OMC, the known concentration of DMF, and the same mass of coffee, extraction and redissolution volumes as in Supplementary Figure 1, the 16-OMC/K content of each sample is calculated following the approach of Schievano et al (2014).

(b) The signal-to-noise ratio (SNR) shown versus the calculated 16-OMC/K content for each original mixture. The value calculated for Arabica sample 12 with no added robusta is indicated.

(c) An expansion of the low SNR region of panel (b), showing the limits of detection and quantitation (LOD, LOQ).

Supplementary Figure 8

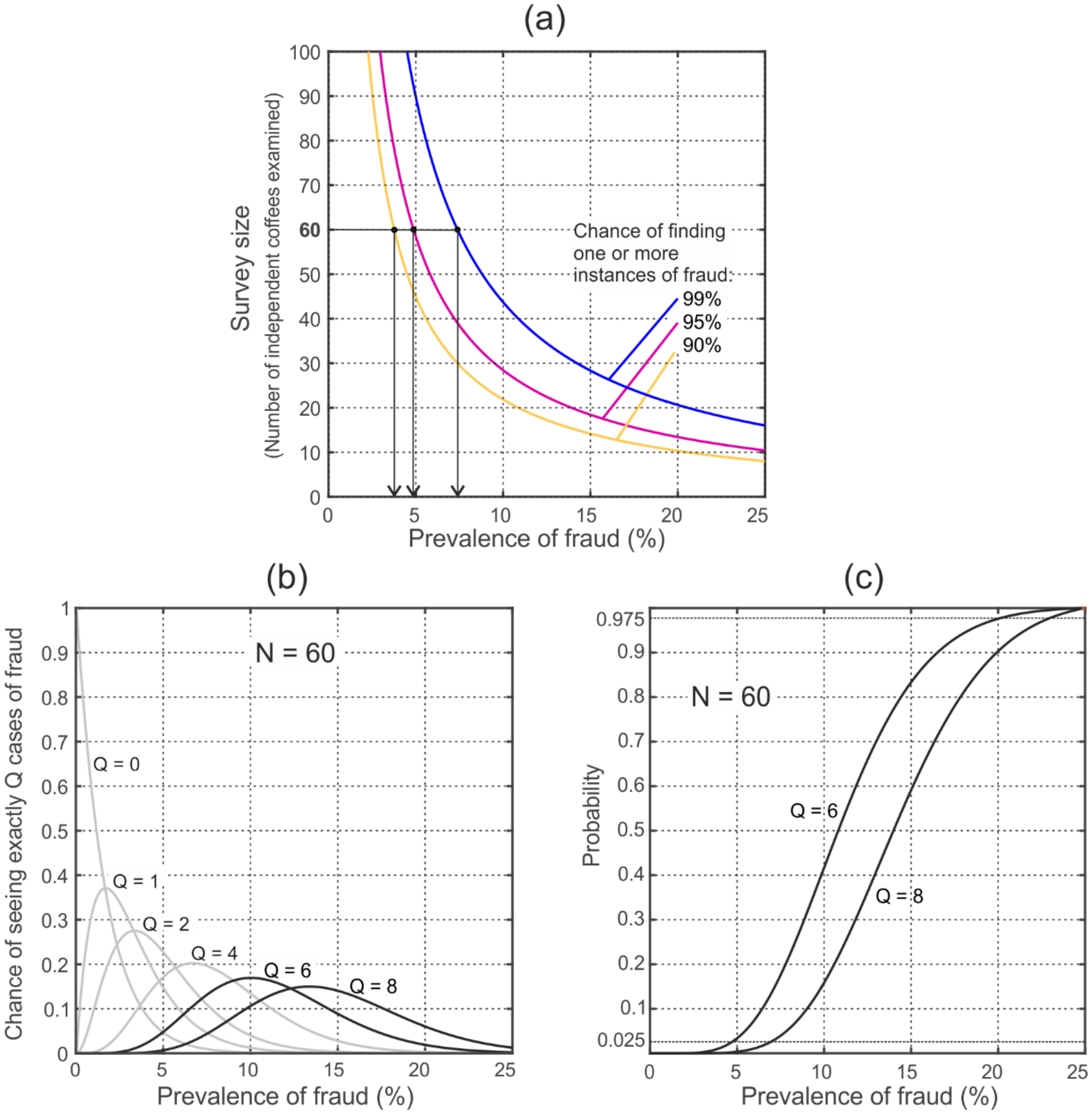

(a) Planning the survey. The graph shows the number of samples that need to be examined as a function of the (*a priori* unknown) fraud prevalence, in order to achieve 99%, 95% or 90% chance of seeing at least one instance of fraud. If cases of fraud are discovered by the surveillance study, an estimate can be made *a posteriori* of the likely fraud prevalence. The functions in (b) show the relative chance of obtaining exactly Q cases of fraud at a range of prevalence rates. Panel (c) shows the cumulative distribution functions for cases Q=6 and Q=8, for which the estimated 95% confidence intervals for the fraud prevalence are 5 - 20% (Q=6) and 7 - 23% (Q=8).
